# The Composition and Functional Capacities of Saliva Microbiota Differ Between Children With Low and High Sweet Treat Consumption

**DOI:** 10.3389/fnut.2022.864687

**Published:** 2022-04-25

**Authors:** Sohvi Lommi, Muhammed Manzoor, Elina Engberg, Nitin Agrawal, Timo A. Lakka, Jukka Leinonen, Kaija-Leena Kolho, Heli Viljakainen

**Affiliations:** ^1^Department of Public Health, University of Helsinki, Helsinki, Finland; ^2^Folkhälsan Research Center, Helsinki, Finland; ^3^Department of Oral and Maxillofacial Diseases, Faculty of Medicine, University of Helsinki, Helsinki, Finland; ^4^Department of Psychology and Logopedics, Faculty of Medicine, University of Helsinki, Helsinki, Finland; ^5^Faculty of Medicine, University of Helsinki, Helsinki, Finland; ^6^Institute of Biomedicine, School of Medicine, University of Eastern Finland, Kuopio, Finland; ^7^Department of Clinical Physiology and Nuclear Medicine, Kuopio University Hospital, Kuopio, Finland; ^8^Foundation for Research in Health Exercise and Nutrition, Kuopio Research Institute of Exercise Medicine, Kuopio, Finland; ^9^Department of Clinical Dentistry, Faculty of Health Sciences, UiT The Arctic University of Norway, Tromsø, Norway; ^10^Children’s Hospital, University of Helsinki and Helsinki University Hospital (HUS), Helsinki, Finland; ^11^Faculty of Medicine and Health Technology, Tampere University, Tampere, Finland

**Keywords:** oral microbiome, sugar, childhood, adolescence, caries, diet

## Abstract

Excess sugar consumption—common in youth—is associated with poor health. Evidence on the relationship between sugar consumption and the oral microbiome, however, remains scarce and inconclusive. We explored whether the diversity, composition, and functional capacities of saliva microbiota differ based on the consumption of select sugary foods and drinks (“sweet treats”). Using 16S rRNA gene sequencing, we characterized saliva microbiota from 11 to 13-year-old children who participated in the Finnish Health in Teens (Fin-HIT) cohort study. The sample comprised children in the lowest (*n* = 227) and highest (*n* = 226) tertiles of sweet treat consumption. We compared differences in the alpha diversity (Shannon, inverse Simpson, and Chao1 indices), beta diversity (principal coordinates analysis based on Bray–Curtis dissimilarity), and abundance (differentially abundant operational taxonomic units (OTUs) at the genus level) between these low and high consumption groups. We performed PICRUSt2 to predict the metabolic pathways of microbial communities. No differences emerged in the alpha diversity between low and high sweet treat consumption, whereas the beta diversity differed between groups (*p* = 0.001). The abundance of several genera such as *Streptococcus*, *Prevotella*, *Veillonella*, and *Selenomonas* was higher in the high consumption group compared with the low consumption group following false discovery rate correction (*p* < 0.05). Children with high sweet treat consumption exhibited higher proportions of nitrate reduction IV and gondoate biosynthesis pathways compared with the low consumption group (*p* < 0.05). To conclude, sweet treat consumption shapes saliva microbiota. Children who consume a high level of sweet treats exhibited different compositions and metabolic pathways compared with children who consume low levels of sweet treats. Our findings reveal novel insights into the relationship between sugary diets and oral microbiota.

## Introduction

The human microbiome—microbes and their genes inhabiting the body—plays an essential role in regulating health and disease, and is influenced by host conditions and several environmental factors, especially the diet (1). Excess sugar consumption is characteristic of the modern diets of children and adolescents (2–4), and associated with several health risks such as dental caries, obesity, and cardiovascular diseases (5–7). Evidence links the aberrant composition of the gut microbiome to the development of conditions including metabolic diseases such as obesity and diabetes (8), possibly a consequence of sugar consumption that may affect the gut habitat leading to altered bacterial communities and influencing metabolism (9). The majority of the mono- and disaccharides ingested are absorbed in the small intestine (9), and thus do not reach the large intestine unless consumed in large quantities (10), whereas oral bacteria are exposed to easily metabolized ingested sugars for which metabolism leads to harmful end-products such as lactic acid (11). Dental caries is a disease resulting in a net mineral loss of teeth due to the acidity produced by bacterial sugar metabolism (12). Furthermore, the frequent consumption of sugar leads to an increase in acid-producing bacteria in the dental biofilm and further to caries.

The role of sugar in the development of caries is well-known, whereas studies on the relationship between oral microbiota profiles and sugar consumption remain scarce. Additionally, existing evidence on the association between oral microbiota profiles and sugar consumption is inconsistent. In adults, no association was found between sugar consumption and saliva microbiota (13, 14), but, in contrast, in subgingival microbiota frequent sucrose consumption associated with decreased species richness and differences in the beta diversity (15), and some cariogenic bacteria such as *Streptococcus sobrinus* were less abundant among those who consumed fewer free sugars (14). Among 17- to 21-year-olds, saliva microbiota profiles varied according to differences in sucrose intake (16). We found only one study examining associations between sugar consumption and oral microbiota among children. In that study among 11-year-old children, the daily consumption of sugar-sweetened beverages was linked to less diversity and richness in oral microbiota and differences in the bacterial abundance (17).

Oral microbiota harbors the largest microbial community after the gut, consisting of several different microbial communities—that is, the microbial composition varies in different locations in the mouth (18). Saliva microbiota consists of bacteria shed from oral surfaces (19), thus representing different communities without having its own resident microbiota (20). Saliva microbiota has been previously linked to metabolic, autoimmune, and immunodeficient conditions, rendering it an affordable and feasible source for biomarkers (21). Furthermore, saliva microbiota may mirror the caries and periodontitis status of the host, and remains relatively stable in orally and systematically healthy individuals (19). Interestingly, oral bacteria can pass through the gastrointestinal tract to a greater extent than previously thought, possibly colonizing the large intestine even in healthy individuals (18, 22). In some disease states, such as rheumatoid arthritis and colorectal cancer, the flux from the oral cavity to the gut may be more pronounced (22), suggesting that oral microbiota plays a role not only in oral diseases, but in systemic health as well.

This study aims to examine whether saliva microbiota profiles, including the diversity and composition, differ according to the consumption of sugary products (“sweet treats”) among school-aged children. In addition, we aim to explore the functional capacities of saliva microbiota. To our knowledge, this is the first study to examine the associations between sugary products and saliva microbiota and its functional capacities. Thus, our study provides novel evidence about the relationships between saliva microbiota and sugar consumption among school-aged children.

## Materials and Methods

This study utilized material from the Finnish Health in Teens (Fin-HIT) study, a large, geographically diverse cohort consisting of over 11 000 children and adolescents aged 9–12 years old at enrolment. Data were collected in 2013 and 2014 in schools across Finland. A detailed description of the cohort appears elsewhere (23). We previously analyzed saliva samples from 1,000 randomly selected Fin-HIT participants (24). After exclusion based on antibiotic use 3 months prior to saliva sampling, missing values in sweet treat consumption as well as low sequencing depth, 700 children remained in the cohort for further investigation.

### Sweet Treat Consumption

Based on children’s responses on a self-administered food frequency questionnaire, **the sweet treat index** (STI) was calculated to indicate a sum for the weekly consumption frequencies of sweet treats (25). These consisted of chocolate/sweets, ice cream, sweet pastries, biscuits/cookies, sugary juice drinks, and sugary soft drinks. Response options ranged from 0 times a week to 14 times a day. To calculate the STI, the consumption frequencies for each food item were summed. Based on tertiles of the STI, participants were categorized as low (first tertile), medium (second tertile) and high (third tertile) groups. We calculated these separately for girls and boys given their different sweet treat consumption patterns (26). Since we sought to compare microbiota profiles between the extreme ends of sweet treat consumption, we included here only participants in the first and third tertiles, leaving us a sample size of 453 with well-defined groups of sweet treat consumption. The low consumption group included 116 (30.0%) girls with STI ≤ 3.5 and 111 (35.5%) boys with STI ≤ 5.5, and the high consumption group included 125 (32.3%) girls with STI > 8.0 and 101 (32.3%) boys with STI > 10.0.

### Background Information

Children’s age- and sex-specific body mass index z-scores (BMIz) were calculated based on the measured height and weight (27) at baseline. Waist–height ratios (WHtR) were calculated by dividing the waist circumference by height (missing values, *n* = 3). When used as covariates, the three missing WHtR values were replaced with the group mean. Maternal occupation at the time of the child’s birth was used as an indicator of socioeconomic status (SES) obtained from the Medical Birth Register from the National Institute for Health and Welfare (THL) (28), and mothers were categorized as upper-level employees, lower-level employees, manual workers, students, or other. We obtained information on the history of cavitated caries lesions and gingival health from THL’s national Register of Primary Health Care visits, which includes data on dental examinations, to which all Finnish children are invited. The oral health data were available for 324 (71.5%) children. Based on the DMFT index (29) (number of decayed, missing, or filled teeth on permanent dentition; missing values, *n* = 159), 228 (70.4%) children were categorized as having no history of cavitated caries lesions (DMFT = 0) for whom oral health data were available, while 96 (29.6%) children had a history of cavitated caries lesions (DMFT > 0). Based on the community periodontal index of treatment needs (30) (CPITN; missing values, *n* = 159) indicating gingival health status, 112 (34.67%) children were categorized as having good oral hygiene (CPITN = 0) and 212 (65.4%) children had poor oral hygiene (CPITN = 1–2) (missing values, *n* = 159). Oral health was examined within ± 12 months of saliva sampling.

### 16S rRNA Gene Amplicon Sequencing and Bioinformatics Analysis

The saliva sampling procedure and 16S rRNA gene sequencing are detailed elsewhere (24). In brief, children provided an unstimulated saliva sample during the school day using the Oragene^®^ DNA (OG-500) Self-Collection Kit (DNA Genotek Inc., Ottawa, Ontario, Canada). Saliva samples were mixed with a stabilizing reagent within the collection tube and stored at room temperature per the manufacturer’s instructions. After an intensive lysis and mechanical disruption protocol of microbial cells, genomic DNA was extracted using a CMG-1035 saliva kit and Chemagic MSM1 nucleic acid extraction robot (PerkinElmer) (24) and the V3–V4 variable regions of the 16S rRNA gene were amplified with primers [S-D-Bact-0341-b-S-17 (5′ CCTACGGGNGGCWGCAG 3′) and S-D-Bact-0785-a-A-21 (5′ GACTACHVGGGTATCTAATCC 3′)] (31). The Truseq (TS)-tailed 1-step amplification protocol was used to amplify the 16S rRNA gene (32). The concentration of the resulting DNA was measured using the Agilent 2100 Bioanalyzer (Agilent Technologies Inc., Santa Clara, CA, United States). The 2 × 270 bp paired-end sequencing of the PCR amplicons was carried out on the Illumina HiSeq1500 platform (Illumina Inc., San Diego, CA, United States). High-quality sequences were processed on the mothur pipeline (v.1.35.1) and reads were aligned using the Silva 16S rRNA reference database (V119), and clustered at >98% homology to identify the operational taxonomic units (OTUs). The bacterial taxa were recognized at the genus level based on sequencing data from previous Fin-HIT studies (24). We calculated alpha diversity indices (Shannon index, inverse Simpson index, and Chao1 index), as well as the beta diversity using Bray–Curtis distances with the R package “vegan” (R version 1.4.1106, package version 2.5-7).

### Statistical Analyses

We performed the chi-square test to compare the categorical background characteristics and the independent samples *t*-test to compare the continuous background characteristics between low and high sweet treat consumption groups. Results are shown as counts and percentages (%), or as means and standard deviations (SDs). These analyses were performed using the SPSS statistical program, version 26 (IBM Corp., Armonk, NY, United States).

We compared the alpha diversity between the low and high sweet treat consumption groups using the analysis of variance (ANOVA) and covariance (ANCOVA) with the R package “stats” (version 4.0.3), while differences in the microbial community composition (beta diversity) were compared using the permutational multivariate analysis of variance (PERMANOVA; R package “vegan,” version 2.5-7). Results are shown for the crude as well as adjusted models, which we adjusted for sex, age, WHtR, and maternal SES. Sex was previously identified as a major contributor to saliva microbiota (24). Sugar intake may contribute to central obesity (33), leading us to include the WHtR. For visualization, we plotted the beta diversity with the principal coordinates analysis (PCoA) using the Bray–Curtis dissimilarity index. Moreover, we performed a sensitivity analysis in which we compared the alpha and beta diversities in a subgroup of 324 children for whom we had complete data on the oral health variables, and adjusted further for caries status and gingival health status to examine the possible distracting effect of caries and gingivitis. In the entire sample, we identified differentially abundant OTUs at the genus level using general linear models with a negative binomial distribution using the DESeq2 function incorporated in the R package “phyloseq” (version 1.34.0), correcting the *p*-values using the false discovery rate (FDR). These analyses were performed using version 1.4.1106 of the R software program. We set the level of statistical significance to *p* < 0.05.

### Analysis of Metabolic Pathways

We predicted the functional potential of saliva microbiota using the Phylogenetic Investigation of Communities by Reconstruction of Unobserved States (PICRUSt2; version 2.0.0-b.2) (34) and used the MetaCyc database as the pathway reference. We identified the differentially present pathways between groups of low and high sweet treat consumption with STAMP (v.2.1.3) (35) using the Welch’s test applying the Bonferroni correction as well as the FDR-adjusted (Benjamini-Hochberg correction) *p*-value of 0.05.

## Results

### Participant Characteristics

The mean (SD) age of children was 11.7 (±0.3) years, with 53.2% of whom were girls. The majority (98.3%) spoke either Finnish or Swedish (the national languages in Finland) as their native language, with a likely similar ethnic background. Table 1 summarizes the characteristics of participants based on low and high sweet treat consumption. Low and high sweet treat consumption groups exhibited similar background characteristics; they differed modestly based on age (*p* = 0.044), but not based on any other background variable including oral health status (*p* > 0.05).

**TABLE 1:** Participant characteristics by low and high sweet treat consumption (*n* = 453).

	Low	High	*p*
	*n* = 227	*n* = 226	
**Age in years, mean (SD)**	11.65 (± 0.34)	11.72 (± 0.36)	0.044^a^
Missing, *n*	0	0	
**BMI z-score^b^, mean (SD)**	0.11 (± 1.02)	-0.03 (± 1.00)	0.138^a^
Missing, *n*	0	0	
**WHtR^c^, mean (SD)**	0.43 (± 0.04)	0.42 (± 0.04)	0.284^a^
Missing, *n*	3	3	
**Maternal SES^d^, *n* (%)**			0.435^e^
Upper-level employees	103 (45.4%)	94 (41.6%)	
Lower-level employees	81 (35.7%)	89 (39.4%)	
Manual workers	17 (7.5%)	19 (8.4%)	
Students	19 (8.4%)	12 (5.3%)	
Other	7 (3.1%)	12 (5.3%)	
Missing, *n*	0	0	
**Caries status^f^, *n* (%)**			0.627^e^
No	119 (71.7%)	109 (69.0%)	
Yes	47 (28.3%)	49 (31.0%)	
Missing, *n*	61	68	
**Gingival health status^g^, *n* (%)**			0.483^e^
Healthy	54 (32.5%)	58 (36.7%)	
At risk	112 (67.5%)	100 (63.3%)	
Missing, *n*	61	68	

*^a^Results from independent samples t-test.*

*^b^Age- and sex-specific BMI z-scores calculated based on measured height and weight (27).*

*^c^Waist–height ratios calculated by dividing waist circumference by height.*

*^d^Maternal occupation at the time of child’s birth from the Medical Birth Register from the National Institute for Health and Welfare (28).*

*^e^Results from the chi-square test or Fisher’s exact test.*

*^f^Caries status based on dichotomous scores of decayed (D), missing (M) due to caries, and filled (F) permanent teeth (DMFT): no, no history of cavitated caries lesions; yes, history of cavitated caries lesions.*

*^g^Gingival health status based on Community Periodontal Index for Treatment Needs (CPITN) values: healthy = 0, at risk = 1 or 2. BMI, body mass index; SES, socioeconomic status; WHtR, waist–height ratio.*

### Alpha and Beta Diversities

**FIGURE 1:**
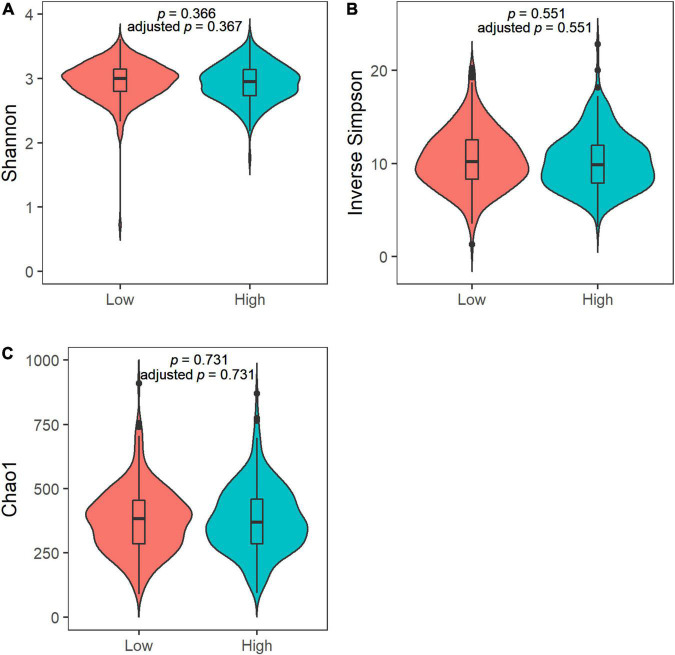
Violin plots of the alpha diversity in the saliva microbiota in children with low (*n* = 227) and high (*n* = 226) sweet treat consumption for **(A)** Shannon index, **(B)** Inverse Simpson index, and **(C)** Chao1 index. Adjusted *p*-value based on an analysis adjusted for sex, age, waist–height ratio, and maternal socioeconomic status. Results from ANOVA and ANCOVA.

**FIGURE 2:**
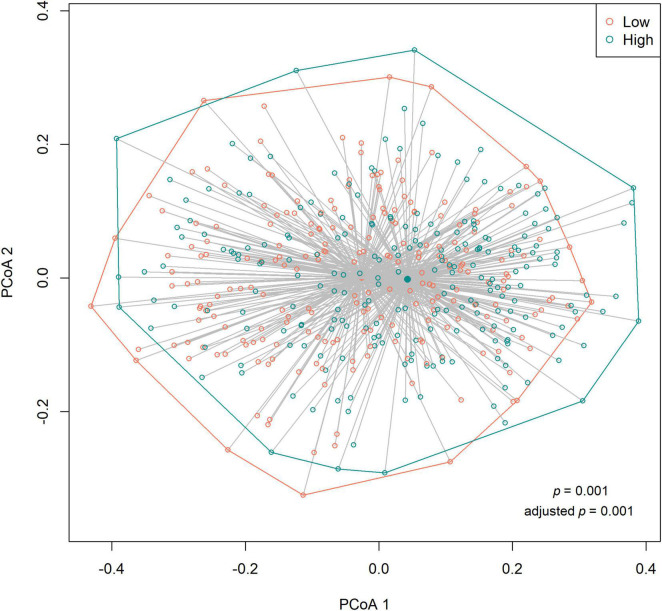
Principal coordinates analysis (PCoA) based on the Bray–Curtis distances (beta diversity) according to low (*n* = 227) and high (*n* = 226) sweet treat consumption. Results based on PERMANOVA. Adjusted *p*-value from a model adjusted for sex, age, waist–height ratio, and maternal socioeconomic status.

We observed no differences in the alpha diversity between low and high sweet treat consumption in terms of the Shannon index, the inverse Simpson index, or the Chao1 index in a crude model nor in a model adjusted for sex, age, WHtR, and maternal SES (*p* > 0.05 for all; Figure 1). In contrast, the beta diversity of saliva microbiota differed between low and high sweet treat consumption in a crude model (*R*^2^ = 0.011, *p* = 0.001) as well as in a model adjusted for age, sex, WHtR, and maternal SES (*R*^2^ = 0.011, *p* = 0.001; Figure 2).

The sensitivity analyses regarding the alpha and beta diversities among children for whom information was available on caries and gingival health status produced similar results as those for the entire sample. The alpha diversity in terms of the Shannon index, the inverse Simpson index, and Chao1 index did not differ between the low and high sweet treat consumption groups (*p* > 0.5 for all; Supplementary Figure 1). The beta diversity differed between the low and high sweet treat consumption groups in a crude model (*R*^2^ = 0.015, *p* = 0.001) and in a model adjusted for sex, age, WHtR, maternal SES, caries status, and gingival health status (*R*^2^ = 0.015, *p* = 0.001; Supplementary Figure 2).

### Differentially Abundant Taxa

The six most abundant phyla in the entire sample were *Firmicutes* (52.2%), *Bacteroidetes* (17.9%), *Proteobacteria* (16.3%), *Actinobacteria* (7.0%), *Candidate division* TM7 (3.5%), and *Fusobacteria* (2.9%), accounting for 99.8% of saliva microbiota in all participants. Since adjusting for covariates did not affect the alpha and beta diversity results, we conducted further abundance analysis without adjustments. We identified differences in the relative abundance of bacteria at the genus level according to sweet treat consumption in a total of 37 OTUs whose abundance differed significantly (Table 2). Compared with the low sweet treat consumption group, we observed a higher abundance of *Veillonella*, *Prevotella*, *Streptococcus*, *Megasphaera*, *Campylobacter*, and *Selenomonas*, and a lower abundance of several genera such as *Haemophilus, Parvimonas*, *Anaerovorax, Treponema*, *Staphylococcus*, and *Fusobacterium* in the high sweet treat consumption group (FDR-adjusted *p* < 0.05; Table 2).

**TABLE 2:** List of differentially abundant OTUs at genus level (or closest taxa) according to sweet treat consumption.

OTU	Nearest taxa	Base mean	Log2fold change	*p*
Otu000002	*Veillonella*	4131.816	+0.486	0.010
Otu000005	*Prevotella*	2265.019	+0.699	0.001
Otu000007	*Micrococcineae*	1921.206	−0.340	0.025
Otu000011	*Streptococcus*	1028.093	+0.407	0.040
Otu000020	*Megasphaera*	423.199	+0.598	0.006
Otu000021	*Campylobacter*	299.759	+0.317	0.032
Otu000022	*Prevotellaceae*	270.280	−0.503	0.016
Otu000031	*Coriobacterineae*	158.380	+0.465	0.008
Otu000032	*Oribacterium*	157.283	−0.272	0.035
Otu000036	*Leptotrichia*	86.499	−0.406	0.030
Otu000039	*Haemophilus*	78.997	−0.974	0.030
Otu000048	*Catonella*	59.865	−0.365	0.009
Otu000050	*Johnsonella*	57.463	−0.502	0.001
Otu000043	*Prevotella*	48.113	+0.614	0.016
Otu000047	*Selenomonas*	46.146	+0.645	0.016
Otu000055	*Prevotella*	39.515	−1.151	1.48E−05
Otu000061	*Candidate division SR1*	39.140	−1.033	0.042
Otu000083	*Parvimonas*	18.304	−0.665	0.025
Otu000100	*Actinomycetales*	13.005	+0.627	0.030
Otu000102	*Prevotella*	11.857	+1.078	0.003
Otu000106	*Candidate division SR1*	10.820	−0.717	0.040
Otu000082	*Prevotellaceae*	10.341	−4.487	0.010
Otu000108	*Peptostreptococcaceae*	9.542	−1.051	8.34E−05
Otu000117	*Peptococcus*	7.913	−0.645	0.028
Otu000123	*Johnsonella*	5.371	−1.430	1.11E−06
Otu000134	*Veillonella*	4.640	−0.694	0.001
Otu000126	*Anaerovorax*	2.997	−4.207	0.001
Otu000154	*Oribacterium*	2.845	−0.471	0.041
Otu000175	*Treponema*	2.246	−1.230	0.009
Otu000174	*Veillonella*	2.063	−0.901	0.005
Otu000190	*Micrococcineae*	1.603	−1.004	0.016
Otu000210	*Prevotella*	1.579	+1.254	0.009
Otu000189	*Staphylococcus*	1.323	−1.137	0.040
Otu000211	*Fusobacterium*	1.125	−0.806	0.037
Otu000232	*Veillonella*	1.061	−0.614	0.042
Otu000240	*Streptococcus*	0.990	−0.627	0.030
Otu000242	*Haemophilus*	0.869	−0.665	0.040

*A positive log2fold change value indicates a higher abundance, while a negative value indicates a lower abundance in the high sweet treat consumption group (n = 227) compared with low consumption (n = 226). P-value adjusted for false discovery rate.*

*Base mean refers to the mean of normalized counts across all samples. OTU, operational taxonomic unit.*

Since adding the oral health variables as covariates in the sensitivity analysis did not change the alpha and beta diversity results, we conducted no further analyses on the abundance or functional capacities in the subsample with the oral health data.

### Functional Capacities of Saliva Microbiota

**FIGURE 3:**
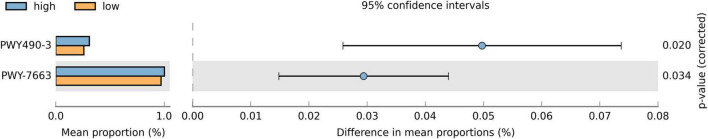
Proportions of differentially active metabolic pathways between children with a low and high sweet treat consumption. Metabolic pathways were predicted using PICRUSt2 and analyzed with STAMP. Differences in mean proportions are shown with 95% confidence intervals. Results based on Welch’s test, *p*-values using the Bonferroni correction. Only pathways with adjusted *p*-value < 0.05 are shown. PWY490-3 = nitrate reduction IV (assimilatory), PWY-7663 = gondoate biosynthesis (anaerobic) from MetaCyc (https://metacyc.org/).

Since the relative abundance of various taxa differed between groups, we studied their overall effects on saliva microbiota. We predicted the functional capacities of the microbiota and, using the Bonferroni correction method, discovered two metabolic pathways (Figure 3) that were more abundant in the high sweet treat compared with the low sweet treat consumption group: assimilatory nitrate reduction IV (adjusted *p* = 0.02) and anaerobic gondoate biosynthesis (adjusted *p* = 0.034). When adjusting the FDR using the Benjamini-Hochberg method, we found 82 metabolic pathways that were differentially present in children with low and high sweet treat consumptions (FDR-adjusted *p* < 0.05). These results appear in Supplementary Figure 3.

## Discussion

In this study of Finnish children aged approximately 12 years old, we found differences in the composition and functional capacities of saliva microbiota between those with low sweet treat consumption and those with high sweet treat consumption. As sweet treats, we considered a range of commonly consumed sugary drinks and foods, reflecting a relevant health-related eating behavior instead of individual food items. The groups did not differ in terms of their oral health. We identified no differences in the alpha diversity between groups, whereas different compositions of saliva microbiota were observed. We also found several genera differentially abundant in the group with a high sweet treat consumption. Therefore, we sought to identify their effects by predicting their functional capacities. We uncovered two metabolic pathways relating to nitrate and gondoate metabolism highlighted in the group with a high sweet treat consumption.

We found no difference in the diversity and richness of saliva microbiota between low and high sweet treat consumption frequencies. In a study among 11-year-old Chinese children, 30 children reporting daily consumption of sugar-sweetened beverages presented with less rich and diverse oral microbiota when compared with 150 children who consumed such beverages less than six times a week (17). By contrast, a study measuring the intake of free sugars among young Danish adults found no difference in the alpha diversity between low (<5% of total energy intake [E%]) and high intake (≥5E%) groups (14). In that study, they measured the amount rather than the frequency of intake, finding that even the high consumption group exhibited a mean intake below the recommendation of no more than 10E% (36). The different measurements of sugar consumption in these studies may partly explain the differing results.

In contrast to the diversity, we found that the composition of microbiota differed according to sweet treat consumption. A relative abundance analysis revealed 37 differentially abundant OTUs, indicating that sugar consumption (measured as a use frequency of sweet treats) is a relevant predictor of the composition of saliva microbiota. We observed OTUs belonging to the core taxa including *Veillonella*, *Prevotella*, and *Streptococcus* (37) enriched in the group with a high sweet treat consumption. Although part of the core microbiota, these genera may associate with oral diseases (38–40). *Veillonella* species are non-motile anaerobic bacteria in the phylum *Firmicutes*, abundantly found in the oral cavity (41). Some oral *Veillonella* species have been associated with the development of caries, periodontitis, peri-implantitis, and other oral diseases (38, 42), and, for example, *V. parvula* associates with severe early childhood caries (43). However, some *Veillonella* species. convert lactic acid to weaker acids, possibly diminishing caries (41). Moreover, they can convert nitrate into nitrite, which is favorable to oral health. As an early colonizer in the oral cavity, *Veillonella* play a critical role in guiding the development of polymicrobial biofilm communities in the oral microenvironment (42). *Prevotella* are among the most dominant and abundant bacteria in the oral cavity with moderately saccharolytic abilities (44). Furthermore, *Prevotella* have been connected to early childhood caries (40), and *P. intermedia* was shown to be characteristic in patients with periodontitis (45).

*Streptococci* form a part of the core microbiota in the oral cavity, with the majority of *Streptococcus* species being acidogenic and/or acid-tolerant (46). Such bacteria have the ability to metabolize carbohydrates through fermentation and produce acids as well as efficiently colonize oral tissues (39). *S. mutans* is a well-known cariogenic species (47), and, while present in a healthy mouth, its abundance increases with the growing availability of sucrose (15). Sucrose increases the biomass of *S. mutans* in the biofilm through an acidic environment and intra- and extracellular polysaccharide synthesis (48), with frequent exposure to dietary sugar acidifying the microenvironment on the teeth leading to enamel demineralization (39). In addition to *S. mutans*, *S. sobrinus* is another species linked to caries (39). By contrast, some *Streptococcus* species can neutralize acidity by producing alkali, as well as producing hydrogen peroxide and antimicrobial compounds (39), thus inhibiting the growth of *S. mutans*. In a healthy mouth, a balance between cariogenic bacteria and non-cariogenic commensal bacteria exists. *Haemophilus* is another common inhabitant of the oral cavity, including *H. parainfluenzae*, which appears to carry some beneficial immunomodulatory effects (49). Then again, *H. influenzae* is a well-known pathogen (50). Here, we noticed a lower abundance of *Haemophilus* in the high sweet treat consumption group compared with the low sweet treat consumption group.

We found *Treponema* and *Staphylococcus* were less abundant in the high sweet treat consumption group compared with the low sweet treat consumption group. *Treponema* has been associated with periodontitis as well as correlating positively with inflammatory cytokines in these patients (51). Although *T. denticola* belongs to the red complex, which is considered the most destructive group of periodontal pathogens (52), and is commonly found in patients with periodontal diseases, other *Treponema* species associate with periodontal diseases as well (53). By contrast, *S. epidermidis* was found enriched in caries-free children (54), and some antibiotic-resistant *S. epidermis* have been identified in the dental plaque of healthy individuals (55). Then, again, pathogenic species *S. aureus* has been linked to several oral diseases such as periodontitis (56). The clinical relevance of these findings remains unclear.

When we predicted the functional capacities of the microbiota, we found two metabolic pathways—nitrate reduction and gondoate biosynthesis—enriched in the group with a high sweet treat consumption. Nitrate metabolism has been associated with a lower occurrence of caries and gingival inflammation, and appears to participate in the maintenance of host oral and systemic health (57). Nitrate (NO_3_^–^) appears to inhibit the salivary acidification resulting from glucose ingestion. Denitrifying oral bacteria reduce nitrate to nitrite (NO_2_^–^) and further to nitric oxide (NO), which has antimicrobial properties and can possibly limit the growth of certain bacteria affecting the composition of the biofilm (57). Nitrite can be reduced to ammonium as well and the presence of nitrate in the oral cavity and is associated with a decrease in the production of lactate and an increase in the production of ammonium. This may result in neutralizing pH and further protect from caries. Moreover, the enterosalivary nitrate–nitrite–nitric oxide pathway depends on oral nitrate-reducing bacteria, and these bacteria contribute to the storage pool of nitrite and nitric oxide in the blood and tissues, consequently positively influencing the host’s physiological status such as blood pressure (58). Dietary sugars, especially in the form of sugar-sweetened beverages, may impact blood pressure (59). Taken together, the nitrate reduction pathway may be upregulated to neutralize acidity resulting from frequent sugar consumption. This may in part explain the similar caries status we found in low and high sweet treat consumption groups. Gondoate is a long-chain unsaturated fatty acid, and its biosynthesis pathway was enriched in the saliva microbiota of adult patients with oral squamous cell carcinoma (60). In the gut microbiome, gondoate biosynthesis decreased during the exacerbation of Crohn’s disease (61). The clinical relevance of our findings that the gondoate pathway upregulates in the high sweet treat consumption group remains unclear.

We adjusted the alpha and beta diversity analyses for age, sex, waist–height ratio, and maternal SES. These potential confounders did not influence the alpha and beta diversities, indicating that sweet treat consumption independently impacts microbial diversity and composition at least in the saliva. In addition, we ran a sensitivity analysis among those children for whom information on their oral health was available (the DMFT index to indicate a history of cavitated caries lesions and CPITN to indicate gingival health), and adjusted the alpha and beta diversity analyses further for these variables. In this study, these were utilized as dichotomous variables, and adjusting for them changed neither the alpha nor the beta diversity results. Given the nature of the dichotomous variables, we were unable to identify those with more severe oral health problems. However, we found some OTUs related to oral infection-associated genera at a higher abundance in the high sweet treat consumption group. Oral health was examined on average 4.3 (±2.2) months prior to or after saliva sampling, but this was unlikely to have impacted these results.

The strengths of the study include the large number of children in the Fin-HIT cohort study conducted across Finland as well as the anthropometric measurements and saliva sampling completed by trained fieldworkers. Moreover, data relating to oral health were obtained from objective and reliable national health care registers. Another strength of our study lies in our use of a summed variable, indicating the consumption of several different types of sugary drinks and foods. Yet, we acknowledge that this measurement was not capable of capturing all possible sources of added sugar. Nonetheless, this study carries some potential limitations as well. There was a lack of standardization in saliva sampling in relation to the time of day or meal, although sampling was carried out primarily in the morning after breakfast, but before lunch. The timing of food intake may influence the diurnal variation of microbiota (62), although microbial profiles were found to remain stable for 24 hours and even for a week (63). We had no information on the putative cases with a low saliva flow rate, possibly impacting microbiota (20). Furthermore, our data do not include information on dental hygiene habits. To overcome this, we considered CPITN as a reflection of such habits. Not assessing smoking represents a minor limitation, given that smoking is extremely rare among Finnish 12-year-old children (64). Food consumption information was assessed through a short, self-administered food frequency questionnaire, and while it is feasible in a large cohort of school-aged children, it does not provide detailed information on the components of the diet. Moreover, misreporting is possible when measuring food consumption. Then, again, food frequency questionnaires rank participants according to their food consumption (65), which we achieved here. Data on saliva microbiota were based on 16S rRNA gene amplicon sequencing and the Silva reference database,^1^ which provide species-level identification of bacteria for only some OTUs. Moreover, we relied on sequencing data from previous Fin-HIT studies (24), and thus, were limited to a genus-level analysis. Species-level identification of the bacteria would have been beneficial. We had no information on total bacterial loads, possibly relevant for this topic (66, 67). In addition, the functionality of microbiota was predicted using PICRUSt2, which provides >80% reliability to pathway discovery (34, 68). Some methodological challenges may be overcome using shot-gun metagenomic approaches instead (69).

To conclude, this study revealed that sweet treat consumption shapes saliva microbiota and its functions in school-aged children. The frequent consumption of sweet treats associated with a differentially abundant microbiota as well as with differentially expressed metabolic pathways. Our findings improve our understanding of the impact of sugary diets on the oral microbiome and can be used to target future studies.

## Data Availability Statement

The datasets presented in this study can be found in online repositories. The names of the repository/repositories and accession number(s) can be found below: EGA; EGAS00001003039.

## Ethics Statement

This study adhered to the Declaration of Helsinki, and the children and one guardian of each child provided their written informed consent before participating. The Ethics Committee of the Hospital District of Helsinki and Uusimaa approved the study protocol (169/13/03/00/10).

## Author Contributions

SL performed the data analysis with assistance from MM and NA, and drafted the manuscript, tables, and figures. All authors contributed to the study design, participated in the data interpretation, revised the manuscript, and accepted the final manuscript for submission.

## Conflict of Interest

The authors declare that the research was conducted in the absence of any commercial or financial relationships that could be construed as a potential conflict of interest.

## Publisher’s Note

All claims expressed in this article are solely those of the authors and do not necessarily represent those of their affiliated organizations, or those of the publisher, the editors and the reviewers. Any product that may be evaluated in this article, or claim that may be made by its manufacturer, is not guaranteed or endorsed by the publisher.

## Footnotes

1
https://www.arb-silva.de/
