# Supplementary material for: The Composition and Functional Capacities of Saliva Microbiota Differ Between Children With Low and High Sweet Treat Consumption
Source: Front Nutr. 2022 Apr 25;9:864687. doi: 10.3389/fnut.2022.864687 (PMC9085455; doi:10.3389/fnut.2022.864687)
Supplement: Supplementary file 1 [file Data_Sheet_1.docx]

Supplementary Material


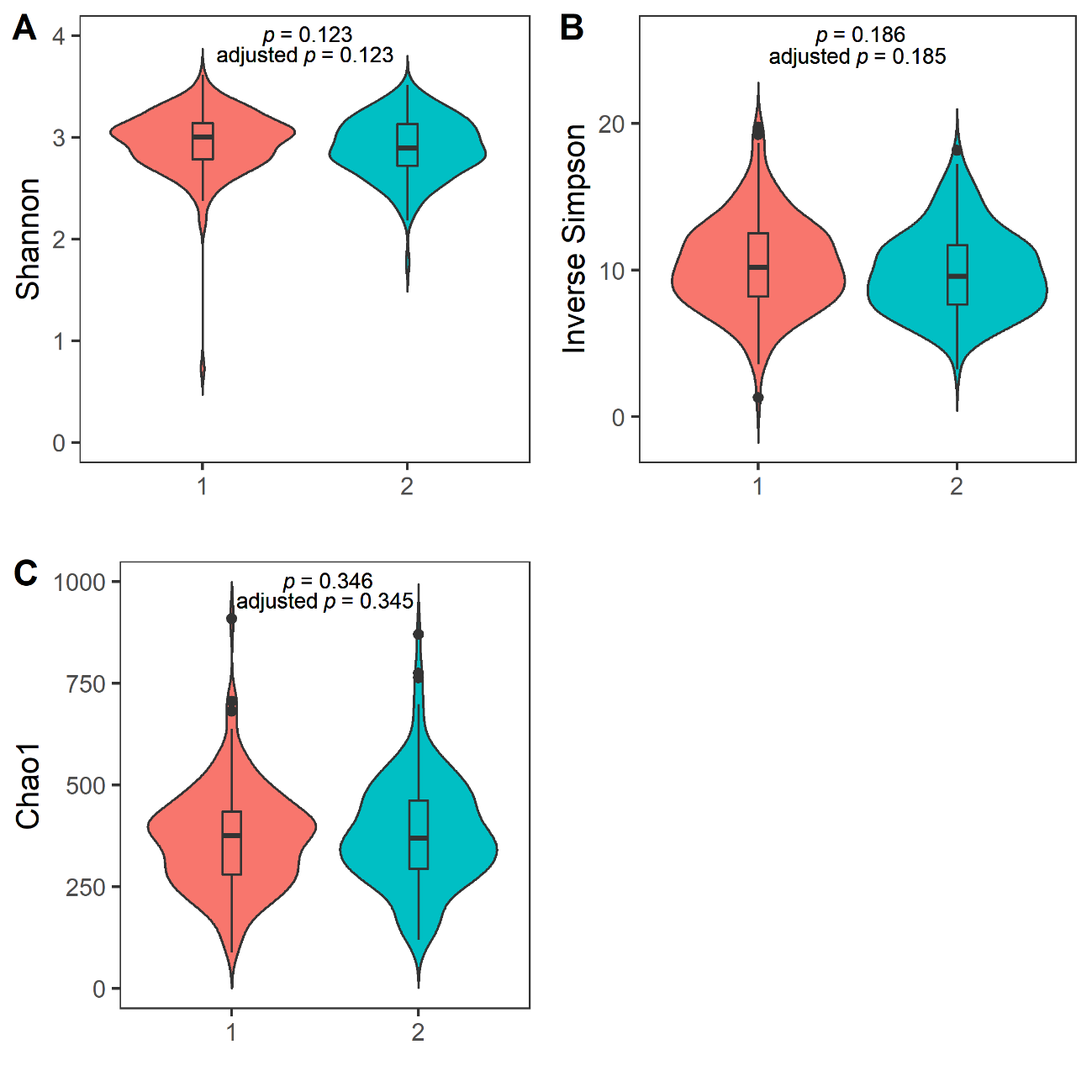


**Supplementary Figure 1** Violin plots of the alpha diversity in the saliva microbiota in children with low (n = 166) and high (n = 158) sweet treat consumption among a subgroup for whom data were available on caries status and gingival health status for the A) Shannon index, B) Inverse Simpson index, and C) Chao1 index. Adjusted *p*-value based on an analysis adjusted for sex, age, waist–height ratio, maternal socioeconomic status, caries status, and gingival health status. Results from ANOVA and ANCOVA.


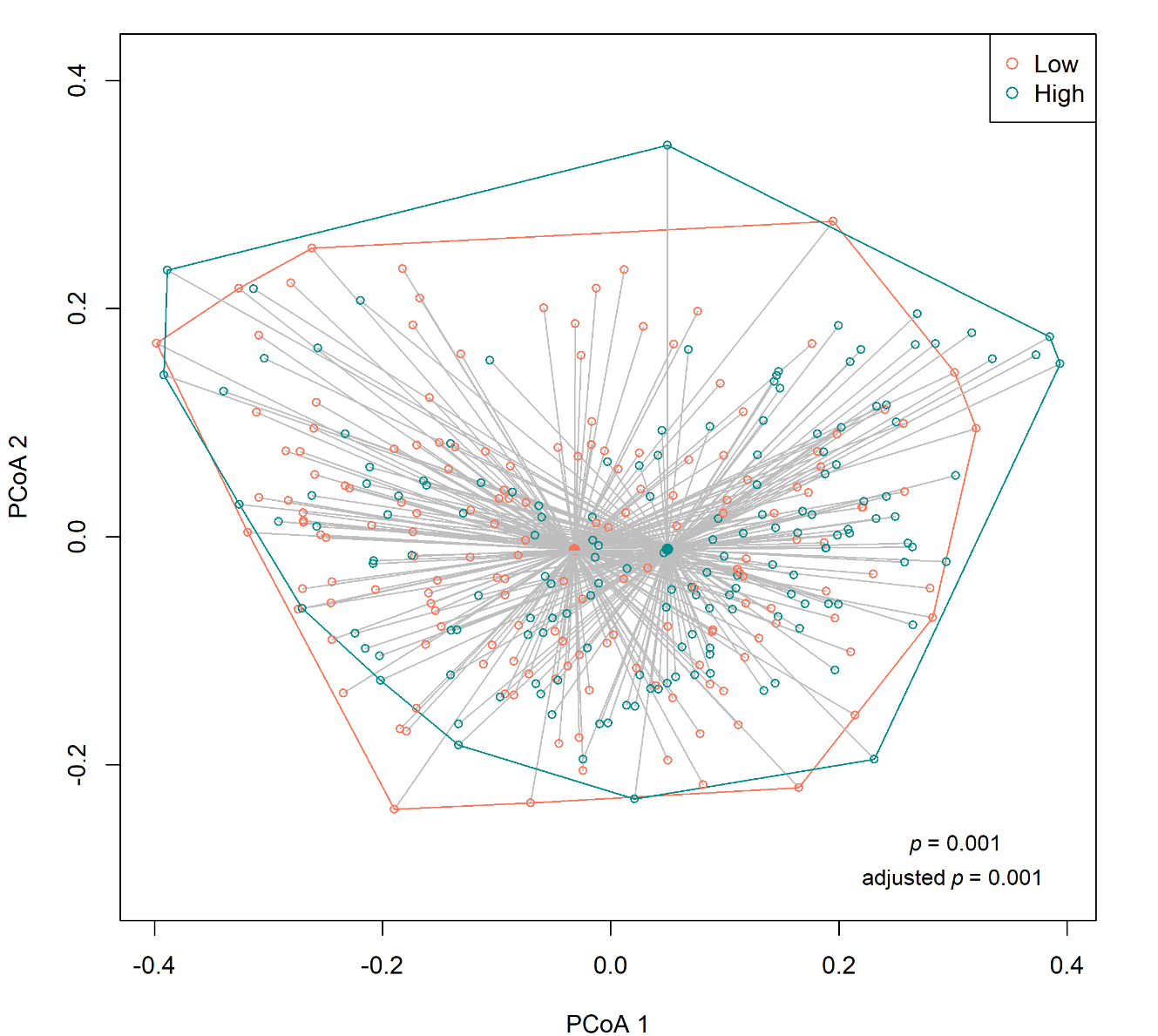


**Supplementary Figure 2** Principal coordinates analysis (PCoA) based on the Bray–Curtis distances (beta diversity) according to low (n = 166) and high (n = 158) sweet treat consumption in a subgroup for whom data were available on caries status and gingival health status. Results from PERMANOVA. Adjusted *p*-value based on a model adjusted for sex, age, waist–height ratio, maternal socioeconomic status, caries status, and gingival health status.


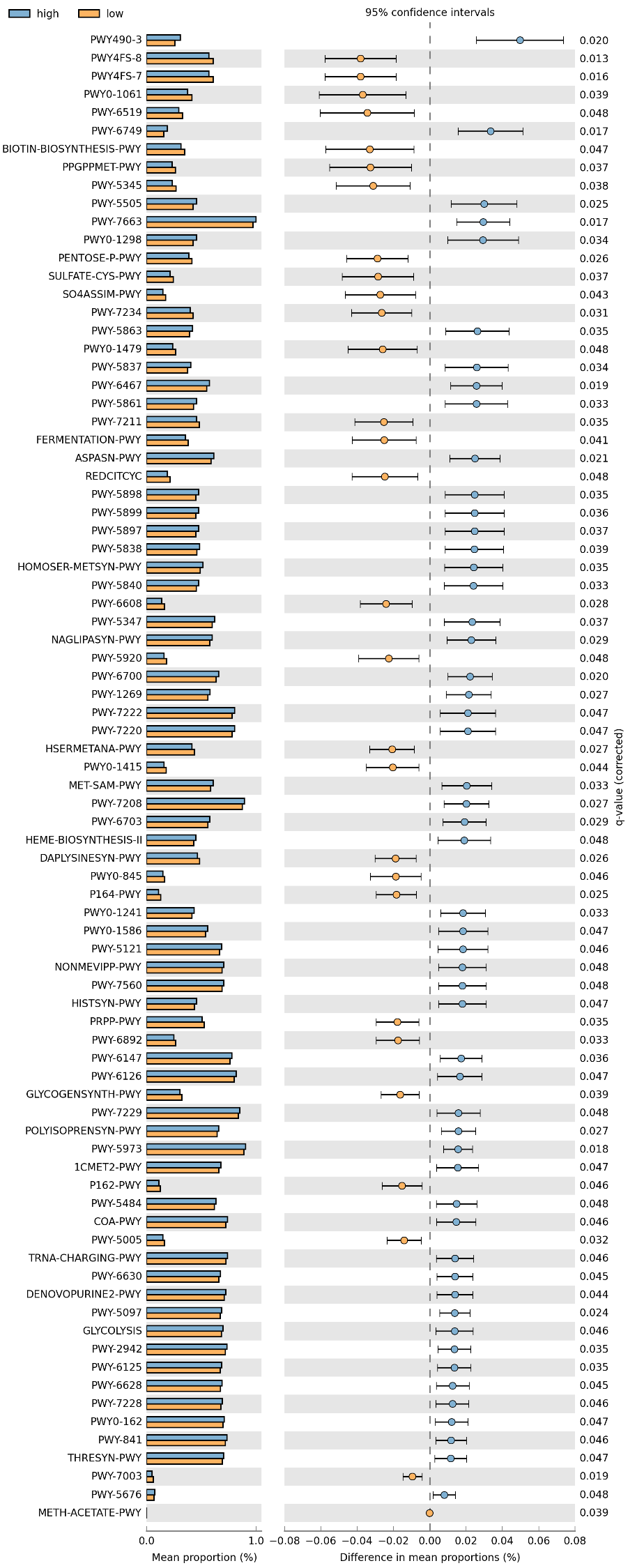


**Supplementary Figure 3** Proportions of differentially active metabolic pathways between children with low and high sweet treat consumption. Metabolic pathways were predicted using PICRUSt2 and analyzed using STAMP. Differences in the mean proportion are shown with 95% confidence intervals. Results based on the Welch’s test adjusted with the false discovery rate (Benjamini-Hochberg correction). Only pathways with corrected *p*-value < 0.05 are shown.
